# Improvement Effect and Regulation Mechanism of Oyster Peptide on Dexamethasone-Induced Osteoporotic Rats

**DOI:** 10.3390/md23090356

**Published:** 2025-09-11

**Authors:** Wei Yang, Wenyu Ma, Xiaoming Qin, Wenhong Cao, Haisheng Lin

**Affiliations:** 1 College of Food Science and Technology, Guangdong Ocean University, Zhanjiang 524088, China; wildperson2004@163.com (W.Y.); wenyudeyouxiang1@163.com (W.M.); cchunlin@163.com (W.C.); haishenglin@163.com (H.L.); 2 National Research and Development Branch Center for Shellfish Processing (Zhanjiang), Zhanjiang 524088, China; 3 Guangdong Provincial Key Laboratory of Aquatic Products Processing and Safety, Guangdong Provincial Engineering Technology Research Center of Seafood, Guangdong Province Engineering Laboratory for Marine Biological Products, Zhanjiang 524088, China; 4 Shenzhen Institute of Guangdong Ocean University, Shenzhen 518120, China; 5 Southern Marine Science and Engineering Guangdong Laboratory (Zhanjiang), Zhanjiang 524000, China

**Keywords:** osteoporosis, dexamethasone, oyster peptides, bone homeostasis, osteogenesis, bone absorption

## Abstract

The increasing global population of the elderly and rising life expectancy have made osteoporosis a more severe public health issue, necessitating the development of safer and more effective therapeutic strategies. This study investigated the osteoprotective effects of low, medium, and high doses of oyster peptide (OP) in dexamethasone (DEX)-induced osteoporotic rats. Pathological analysis showed that OP treatment effectively mitigated bone loss and repaired bone microarchitecture deterioration caused by DEX administration. In the OP groups, levels of the osteogenic markers osteocalcin (OCN) and osteoprotegerin (OPG) were significantly higher than in the DEX group. Moreover, levels of the osteoclastic markers RANKL, Cathepsin K (Cath-K), MMP-9, C-terminal telopeptide of type I collagen (CTX-1), and Deoxypyridine (DPD) were significantly lower. Bone proteomic analysis of the DEX and OP groups revealed that differentially expressed proteins were significantly enriched in pathways related to extracellular matrix and structural reorganization, ECM–receptor interaction, and PI3K-Akt signaling. Furthermore, virtual screening simulations indicated that peptides with lengths ranging from 11 to 20 amino acid residues were involved in modulating the activity of key receptors in these pathways, including Integrins α5β1, Integrins αvβ3, and EGFR. Collectively, these results demonstrate the significant potential of OP as a novel therapeutic agent for osteoporosis.

## 1. Introduction

The global prevalence of osteoporosis is currently 18.3% (23.1% in females and 11.7% in males), making it one of the most significant public health challenges globally [1]. Clinically, it is broadly categorized into primary and secondary types based on etiology. Primary osteoporosis is predominantly age-related, with postmenopausal osteoporosis being the most common form, driven by reduced estrogen secretion after menopause [2]. Secondary osteoporosis stems from various factors, including underlying diseases, medical treatments, prolonged glucocorticoid use, and unhealthy dietary and lifestyle habits [1]. Additionally, idiopathic osteoporosis, characterized by an unknown etiology, is more frequently observed in adolescents and men [3,4].

Bone homeostasis critically relies on the dynamic balance between osteoclastic bone resorption and osteoblastic bone formation. Disruption of this delicate equilibrium leads to microarchitectural deterioration and increased bone fragility, significantly elevating the risk of fracture [5]. While non-pharmacological interventions, such as dietary improvements, increased vitamin D and calcium intake, reduced alcohol and tobacco consumption, and enhanced exercise, are crucial strategies for managing osteoporosis progression, they often prove insufficient for patients in moderate-to-advanced stages. Furthermore, long-term pharmacological treatments, including antiresorptive drugs (e.g., bisphosphonates, estrogen, calcitriol) and anabolic agents (e.g., teriparatide, romosozumab), are frequently associated with diverse side effects, such as gastrointestinal disturbances, as well as hepatic/renal or cardiovascular damage [6]. Consequently, the development of effective and safer strategies for osteoporosis prevention and treatment, particularly those with fewer adverse effects, is of paramount significance for improving patients’ quality of life. In this context, dietary supplementation with natural food-derived ingredients, such as polyphenols [4] and bioactive peptides [7], presents a promising and cost-effective approach, potentially mitigating the risk of osteoporosis through mechanisms including antioxidant, anti-inflammatory, and immunomodulatory effects [8,9].

The unique marine environment and its rich biodiversity serve as an excellent reservoir of novel bioactive substances, with evidence suggesting that high seafood intake correlates with a reduced risk of osteoporosis [10]. Specifically, bioactive peptides derived from various marine sources, including microalgae, fish, mollusks, and crustaceans, have demonstrated promising anti-osteoporotic activities [11]. Among these, oysters (*Ostrea rivularis* Gould), widely cultivated along the coastal regions of China, are highly valued for their exceptional nutritional and medicinal properties [12]. Traditional Chinese Medicine (TCM) has long utilized calcined oysters in formulations such as Longmu Zhuanggu Granules [13] and Gusongbao Granules [14], to enhance calcium levels and manage bone-related ailments. Furthermore, Ejiao Qianggu oral liquid, also containing oysters, has been shown to promote osteoblast proliferation in a dose-dependent manner for osteoporosis treatment [15]. However, the efficacy of these oyster-based TCM formulations is often influenced by complex factors such as raw material origin, processing methods, and compatibility. Crucially, the precise identification and standardization of their active chemical components remain challenging, thereby limiting the broader and evidence-based application of oysters in modern osteoporosis therapy.

Numerous studies have explored the potential anti-osteoporotic components of oysters. For instance, in a mouse model of protein malnutrition, taurine from oyster peptide (OP) has been shown to promote bone growth by upregulating IGF-1 expression and inducing the phosphorylation of JAK2-STAT5 [16]. Furthermore, exosomes isolated from the oyster mantle have demonstrated potent anti-osteoporotic activity by enhancing osteoblast survival through PI3K-Akt signaling and suppressing osteoclast activity via the NF-κB pathway [10]. However, these studies do not fully elucidate the comprehensive anti-osteoporotic potential of oysters. 

The main component of oyster meat is protein, and its bioactive hydrolysates have also been shown to possess therapeutic potential against osteoporosis [12]. Therefore, this study was designed to prepare OP from oyster meat and to investigate their anti-osteoporotic activity. We characterized the prepared OP and evaluated their in vivo anti-osteoporosis efficacy using a rat model of dexamethasone (DEX)-induced osteoporosis. Specifically, we investigated the anti-osteoporotic mechanism and potential targets of OP through histological analysis, bone formation assays, and proteomics. We further used molecular docking to identify the specific peptides within OP that exhibit high anti-osteoporotic activity. This work will lay the foundation for future research and the high-value utilization of OP.

## 2. Results

### 2.1. Composition Analysis of OP

**Figure 1:**
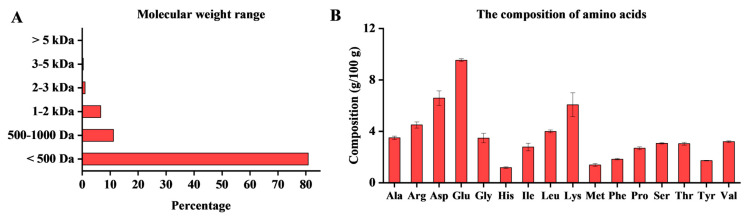
Composition analysis of OP: (**A**) The molecular weight distribution and (**B**) amino acid composition of OP.

Studies have demonstrated that low-molecular-weight bioactive peptides often possess potent anti-osteoporotic activity [7]. In this study, the total protein content of the OP was determined to be 73.10% by the Kjeldahl method. As shown in Figure 1A, size-exclusion chromatography revealed the weight-average molecular weight of OP was 390.4 Da, with the major fraction (80.83%) distributed between 180 and 500 Da. This indicates that our industrialized enzymatic hydrolysis process is highly effective at liberating low-molecular-weight bioactive peptides from oyster meat. The biological activity of peptides is heavily influenced by their amino acid composition. As detailed in Figure 1B, OP contains a rich and diverse profile of amino acids, with 22.28 g of essential amino acids per 100 g. The most abundant amino acids were glutamic acid (9.54 g/100 g), aspartic acid (6.58 g/100 g), and lysine (6.07 g/100 g). The presence of high contents of aromatic, charged amino acids and methionine is particularly significant, as these residues are crucial for moderating the anti-osteoporotic activity of peptides [17].

### 2.2. Effects of OP on Body Weight and Organs

**Figure 2:**
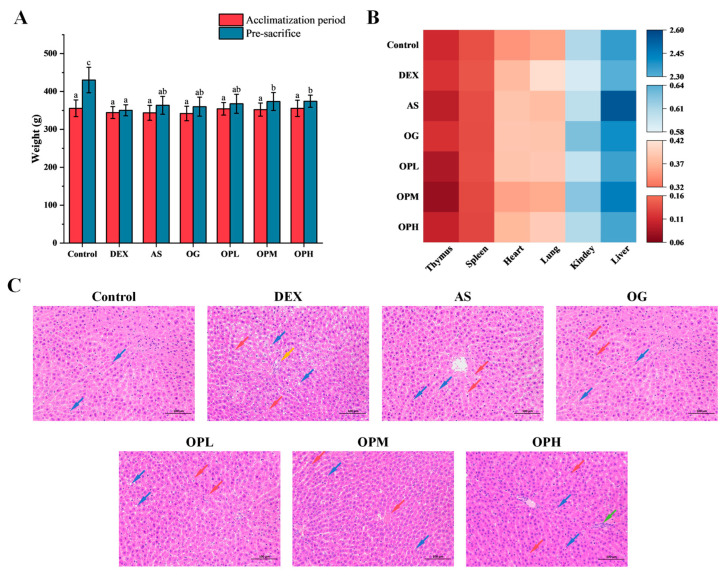
Effects of AS, OG, and OP on (**A**) body weight, (**B**) organ indices, and (**C**) liver morphology in DEX-induced osteoporotic rats. Different letters above the bars in the column chart indicate statistically significant differences in the data. Arrows indicating hepatocellular steatosis (blue), congestion and dilation of hepatic sinusoids (red), hepatocellular necrosis (yellow), and inflammatory cell infiltration (green).

To comprehensively evaluate the systemic effects of the treatments, we monitored the body weight of the rats weekly and calculated their organ indices (Figure 2, Table S1). As shown in Figure 2A, rats in the DEX group experienced a significant decrease in body weight (350.46 ± 11.23 g) compared to the control group (430.23 ± 15.67 g). Treatment with medium and high doses of OP (OPM and OPH, respectively) effectively mitigated this weight loss, resulting in final body weights of 373.64 ± 9.87 g and 374.40 ± 10.12 g, respectively. Analysis of organ indices revealed a systemic impact of DEX (Figure 2B). Relative to the control group, the DEX group showed an increasing trend in the indices of the thymus, spleen, heart, and lungs, while the kidney and liver indices trended downward. This suggests that the observed weight loss was associated with DEX-induced systemic metabolic disorders. Notably, only the alendronate sodium (AS) group exhibited a significant increase in the liver indices compared to the DEX group, potentially indicating the hepatotoxic effects of this Western medication. 

Histopathological examination of liver tissue via H&E staining (Figure 2C) further supported these findings. The DEX group displayed noticeable fat accumulation and liver damage, accompanied by an increasing risk of local necrosis. Both the *Gushukang granules* (osteoporosis granules, OG) and OP groups showed improved fat accumulation and hepatic sinusoid dilation in the liver tissue, suggesting a protective effect against DEX-induced liver damage. However, the OPH intake caused localized hepatic inflammation, which may be attributable to an increased metabolic load on the liver due to the high peptide intake. Overall, the administration of OP within the tested dosage range (300–2700 mg/kg) did not cause any severe adverse effects on the rats’ organs.

### 2.3. Protective Effect of OP Against Bone Loss

**Figure 3:**
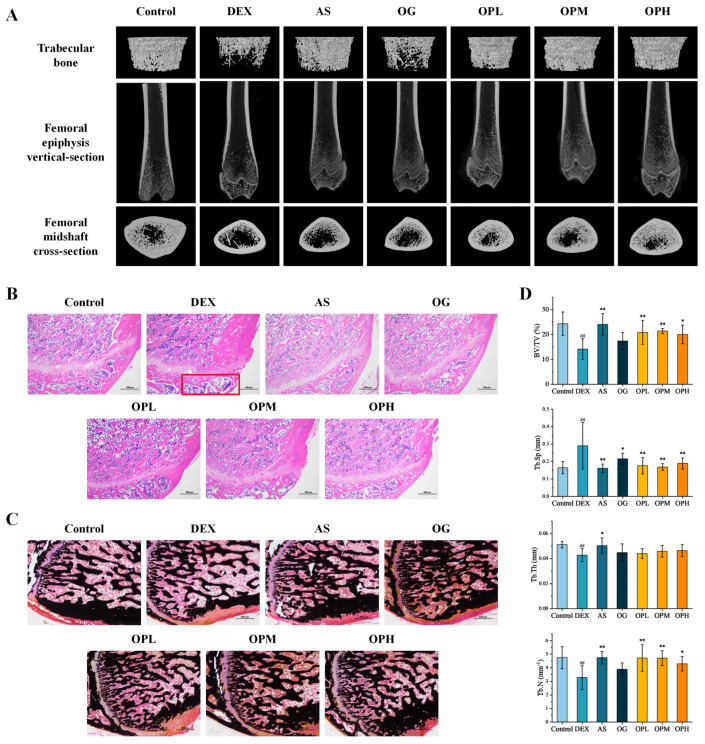
Protective effects of AS, OG, and OP against DEX-induced bone loss in rats: (**A**) Representative micro-CT images displaying vertical and cross-sectional views of the trabecular microarchitecture in rat femurs. Representative staining images of rat femurs for (**B**) H&E and (**C**) Von Kossa. (**D**) Quantitative analysis of bone volume fraction (BV/TV, %), trabecular separation (Tb.Sp, mm), trabecular thickness (Tb.Th, mm), and trabecular number (Tb.N, 1/mm). Data are expressed as the mean ± SEM; ^#^ *p* < 0.05, ^##^ *p* < 0.01, compared with the control group; * *p* < 0.05, ** *p* < 0.01, compared with the DEX group.

The osteoprotective effect of OP was investigated using a DEX-induced osteoporosis model in rats. After 9 weeks of DEX treatment, micro-CT scanning and 3D reconstruction revealed a significant reduction in femoral bone volume and trabecular number in the DEX group compared to the control, indicating severe bone loss (Figure 3A). Both the OP and drug (AS and OG) treatments effectively ameliorated these DEX-induced changes in bone microstructure. Quantitative analysis of the bone microstructure further confirmed these findings (Figure 3D). After 9 weeks of DEX treatment, femoral bone mineral density (BMD) decreased by 41.98%. Treatment with the AS and OG increased BMD by 70.30% and 20.12%, respectively. OP treatment exhibited a protective effect that was intermediate to those of AS and OG. Notably, the low and medium doses of OP were particularly effective, increasing bone volume/total volume (BV/TV) by 47.51% and 51.49%, respectively. Further results for trabecular separation (Tb.Sp), trabecular thickness (Tb.Th), and trabecular number (Tb.N) all indicated that long-term DEX treatment led to significant trabecular bone loss, which was effectively ameliorated by medium and low doses of OP.

Histopathological analysis provided further evidence of OP’s protective effect on bone. The H&E staining results showed that the trabecular bone area in the DEX group was reduced compared to the control, a condition that was alleviated by OP treatment (Figure 3B). Furthermore, to evaluate new bone formation, Von Kossa staining was performed. It revealed that while DEX significantly inhibited new bone formation in the rat femurs, both positive drug controls and OP treatments effectively improved this process (Figure 3C). Overall, these findings demonstrate that OP can effectively mitigate osteoporosis caused by long-term DEX treatment, with a comparable protective effect to that of the positive drug controls.

### 2.4. OP Ameliorates Bone Loss by Promoting Bone Formation

**Figure 4:**
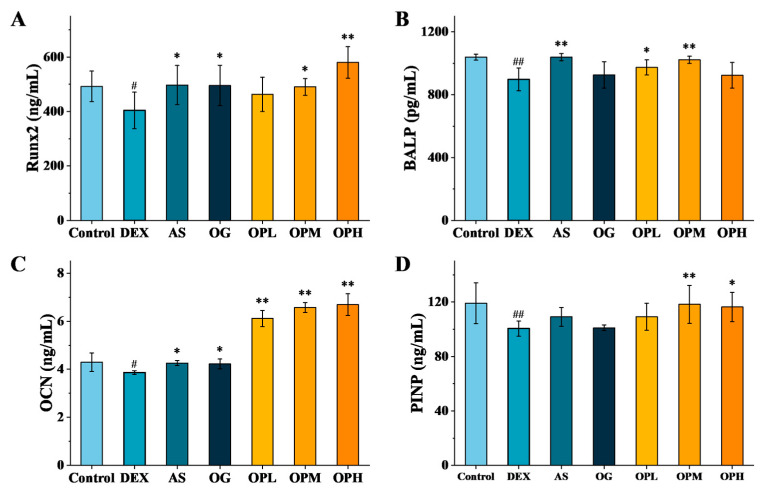
Analysis of bone formation-related indices: (**A**) Runx2, (**B**) BALP, (**C**) OCN, and (**D**) PINP; ^#^ *p* < 0.05, ^##^ *p* < 0.01, compared with the control group; * *p* < 0.05, ** *p* < 0.01, compared with the DEX group.

The dynamic balance between osteoblast-mediated bone formation and osteoclast-mediated bone resorption is essential for bone homeostasis. When this balance is disrupted, it manifests as bone loss, which can lead to osteoporosis [18]. In this study, we investigated the effects of OP on key osteogenic markers (Figure 4). Compared with the control group, the DEX group exhibited a significant downregulation of runt-related transcription factor 2 (Runx2), accompanied by decreased expression of osteocalcin (OCN) and N-terminal propeptides of procollagen type Ⅰ (PINP) and reduced activity of bone alkaline phosphatase (BALP). This indicates that DEX inhibits osteoblast differentiation and maturation by targeting Runx2, thereby reducing bone mass accumulation. Both positive drugs, AS and OG, alleviated the DEX-induced downregulation of Runx2. However, OG did not significantly increase BALP activity or PINP expression, demonstrating its weaker osteoprotective effect compared to AS, and this is consistent with our aforementioned findings. Notably, OP upregulated Runx2 expression in a concentration-dependent manner. At the medium dose (900 mg/kg/d), OP showed a more pronounced promoting effect on BALP activity and on OCN and PINP expression than the positive drug AS. These data collectively indicate that OP can effectively ameliorate osteoporosis caused by long-term DEX treatment by promoting bone formation.

### 2.5. OP Ameliorates Bone Loss by Inhibiting Bone Resorption

**Figure 5:**
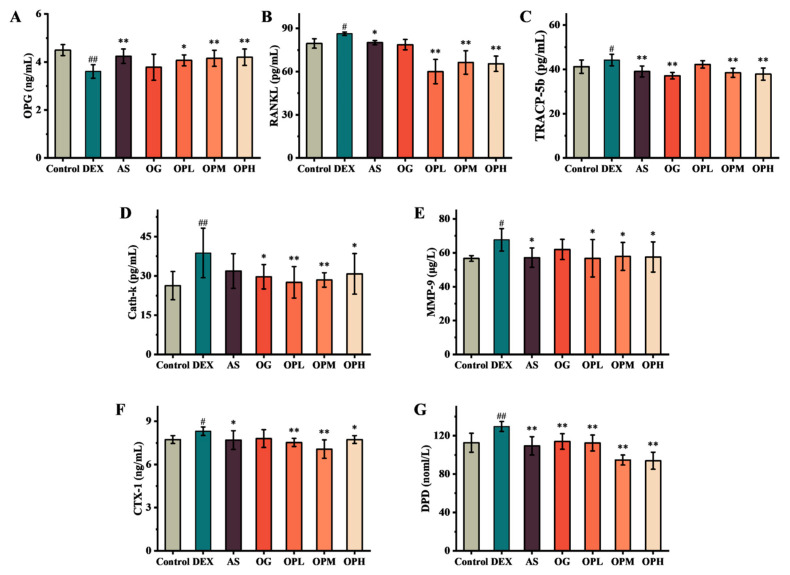
Analysis of bone absorption-related indices: (**A**) OPG, (**B**) RANKL, (**C**) TRACP-5b, (**D**) Cath-k, (**E**) MMP-9, (**F**) CTX-1, and (**G**) DPD; ^#^ *p* < 0.05, ^##^ *p* < 0.01, compared with the control group; * *p* < 0.05, ** *p* < 0.01, compared with the DEX group.

RANK and RANKL are key regulators of osteoclast formation and bone resorption, and their overexpression is a major cause of osteoporosis [19]. Compared to the control group, long-term administration of DEX in rats resulted in a significant downregulation of OPG expression. This weakened the inhibitory effect on the RANKL signaling pathway, thereby promoting osteoclast differentiation (Figure 5A,B). Consequently, the overexpression of Tartrate-Resistant Acid Phosphatase 5b (TRACP-5b), MMP-9, and Cathepsin K (Cath-K) in osteoclasts promoted the degradation of the bone matrix and the destruction of the collagen network (Figure 5C–E). These effects led to elevated blood levels of C-terminal telopeptide of type I collagen (CTX-1) and Deoxypyridine (DPD) (Figure 5F,G). Upon treatment with the positive drug AS, osteoprotegerin (OPG) expression returned to normal, which inhibited the RANKL signaling pathway and reduced the expression of the aforementioned bone resorption markers. In contrast, while the positive drug OG also inhibited the expression of these markers in DEX-treated rats, it did not significantly increase OPG expression. This suggests that OG may inhibit RANKL signaling-mediated bone resorption through a different mechanism. All doses of OP showed effects similar to those of AS. Interestingly, compared to other concentrations, the low-dose OP (300 mg/kg/d) inhibited RANKL expression to a greater extent but did not show superior performance in bone resorption indicators like TRACP-5b and DPD. Similarly, the high-dose OP (2700 mg/kg/d) did not demonstrate better effects on bone resorption markers compared to the medium-dose OP (900 mg/kg/d). Therefore, while all doses of OP demonstrated an anti-osteoporotic effect by inhibiting bone resorption, a comprehensive consideration of the BV/TV index and bone formation markers suggests that the medium dose of OP offers superior efficacy and cost-effectiveness.

### 2.6. Screening of Potential Anti-Osteoporotic Active Peptides

**Figure 6:**
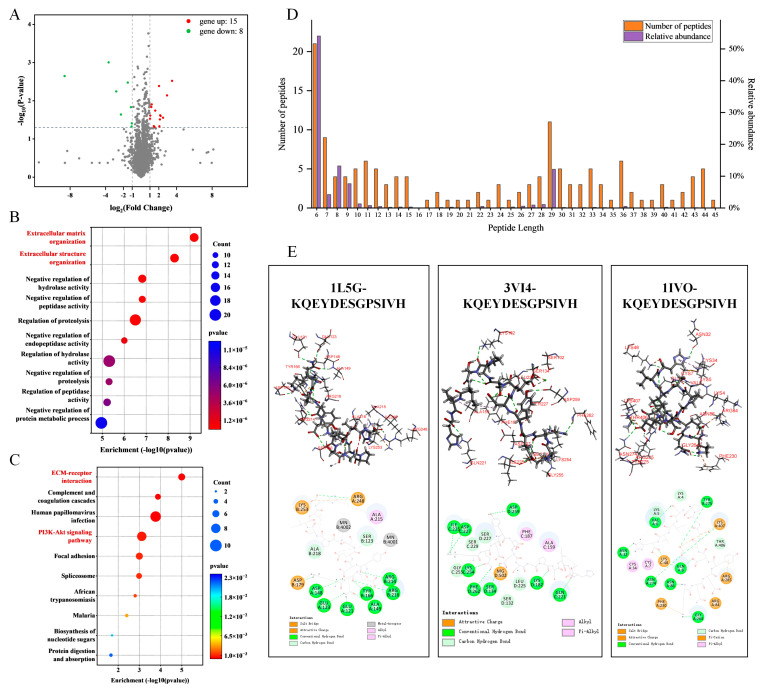
Screening of potential anti-osteoporotic active peptides: (**A**) Volcano plot of differentially expressed genes in the OPH group versus the DEX group (fold change > 2 and adjusted *p*-value < 0.05). (**B**) GO biological process analysis of differentially expressed genes. (**C**) KEGG pathway analysis of differentially expressed genes. (**D**) Polypeptide distribution of OP. (**E**) Visualization of molecular docking between the polypeptide KQEYDESGPSOVH and receptors Integrins α5β1 (1L5G), Integrins αvβ3 (3VI4), and EGFR (1IVO).

To further elucidate the protective effects of OP on DEX-induced osteoporosis, a Data-Independent Acquisition (DIA) proteomics analysis was performed, comparing the DEX group with the OPH group. This analysis identified 15 upregulated and 8 downregulated protein genes (fold change > 2, *p* < 0.05; Figure 6A). GO enrichment analysis of these differentially expressed genes in bone tissue revealed a strong enrichment in biological processes related to extracellular matrix organization and structural reorganization (Figure 6B). Correspondingly, KEGG pathway analysis highlighted that the extracellular matrix–receptor interaction and the PI3K-Akt signaling pathway were significantly associated with the amelioration of DEX-induced osteoporosis by OP (Figure 6C). As key regulators of both osteoblast and osteoclast formation, the PI3K-Akt and RANKL signaling pathways are co-regulated by Integrin and EGFR signals [20,21].

To identify the specific anti-osteoporotic peptides, we performed peptidomics on the oyster hydrolysates and identified 142 peptides (Figure 6D). We then used molecular docking to screen these peptides for their potential binding affinity to key osteoporosis-related receptors, including Integrins α5β1, Integrins αvβ3, and Epidermal Growth Factor Receptor (EGFR) (Table S3). While most high-abundance peptides were 6–10 or 27–29 amino acid residues in length, peptides in the 11–20-residue range exhibited the strongest receptor-binding activity. These highly active peptides were also notably rich in hydrophilic amino acids and lacked aromatic residues (Table 1). Taking the 11-residue peptide KQEYDESGPSIVH as an example, its binding to Integrins α5β1 and EGFR was found to be mediated primarily by salt bridges and hydrogen bonds, while its interaction with Integrins αvβ3 was mainly dependent on hydrogen bonds (Figure 6E). These results collectively suggest that the anti-osteoporotic activity of OP may originate from less abundant but highly active peptides with a specific length and amino acid composition.

## 3. Discussion

Oysters, traditionally regarded as a food–medicine homology, have a long history of use in remedies for bone health. Recent interest has grown in the osteoprotective benefits of their hydrolyzed polypeptides, although the underlying mechanisms have not been fully elucidated [12,22,23,24]. Our findings revealed that oral administration of OP effectively mitigates DEX-induced osteoporosis by modulating bone homeostasis. Specifically, OP promotes osteoblast differentiation through upregulation of the Runx2 pathway and inhibits bone resorption by suppressing the RANKL signaling pathway. This dual regulatory action successfully attenuates DEX-induced bone loss. Furthermore, we utilized proteomics to identify three key osteoporosis targets (Integrins α5β1, Integrins αvβ3, and EGFR). Through molecular docking, we discovered that the anti-osteoporotic effects of OP primarily originate from its low-abundance peptide chains, which are 11–20 amino acid residues in length. Our research not only refines the established osteoprotective mechanisms of OP but also proposes a novel strategy for the prevention and treatment of osteoporosis.

While previous studies have shown that unhydrolyzed oyster matrix proteins can exhibit anti-osteoporotic activity in vivo [24], hydrolysis is generally preferred for producing defined bioactive peptides. This is due to the inherent variability in peptide types and concentrations generated from in vivo protein digestion, which can be influenced by individual physiological states [25]. In general, lower-molecular-weight peptides resulting from hydrolysis tend to exhibit more potent anti-osteoporotic activity [7]. Therefore, the lower average molecular weight of peptides produced via our standardized industrial process, compared to traditional laboratory methods, is likely to translate into enhanced intestinal absorption and superior bioactivity [26]. The compositional analysis of our prepared OP further supports this conclusion, revealing a rich abundance of essential amino acids such as Glu, Asp, Lys, Arg, Leu, and Ala, all of which are known to be crucial for bone formation and mineralization [24,26]. This amino acid profile further underpins the potent anti-osteoporotic potential of OP [2]. Consistent with the previous reports, our study found that the long-term use of AS, a conventional osteoporosis drug, increased the hepatic metabolic burden, thereby elevating the risk of liver disease [6]. In contrast, while the Traditional Chinese Medicine OG demonstrated fewer side effects than Western pharmaceuticals, its therapeutic efficacy was markedly lower. The favorable balance of safety and efficacy observed with OP positions it as a promising candidate for the development of novel osteoprotective health products.

Our in vivo studies strongly support the anti-osteoporotic effects of OP. We found that OP administration significantly improved calcium deposition, thereby enhancing bone volume fraction, a critical indicator of skeletal health [27,28]. Further investigation revealed that OP treatment promoted the expression of key bone formation markers: Runx2, BALP, OCN, and PINP. As a master regulator of osteogenesis, Runx2 integrates signals from multiple pathways, including Wnt, TGF-β, BMP, FGF, and Hedgehog, to orchestrate the expression of critical osteogenic genes [29,30]. The dose-dependent increase in Runx2 expression with OP treatment highlights its positive impact on osteoblast differentiation and maturation. However, concerning bone markers, increasing the OP dosage beyond 900 mg/kg/d showed diminishing returns on OCN and PINP expression, and even reduced BALP expression. While some studies suggest that BALP levels correlate with osteogenic activity [31,32], others indicate increased BALP due to osteoblast apoptosis [33,34]. A plausible interpretation of our results is that elevated serum BALP may be associated with both increased osteoblast apoptosis and activity. The overexpression of Runx2 could potentially prolong the lifespan of senescent osteoblasts, thereby reducing the BALP levels measured in serum. A more comprehensive explanation is that osteoblasts’ differentiation, maturation, and activity are not isolated processes but are intricately linked with osteoclast function to maintain bone homeostasis [35]. The precise interplay between these cells warrants further investigation.

Interestingly, we observed that low-dose OP exhibited a more pronounced inhibition of RANKL expression compared to medium and high doses. As a critical factor in osteoclast differentiation and maturation, RANKL activation of the RANK receptor initiates osteoclastogenesis; thus, decreased RANKL expression is expected to suppress osteoclast activity [36]. However, this observation was not consistently reflected in other bone resorption markers, including OPG, TRACP-5b, Cath-K, MMP-9, CTX-1, and DPD, where low-dose OP did not show superior ameliorative effects compared to the medium dose. RANKL and OPG are both secreted by osteoblasts, and their ratio (OPG/RANKL) is crucial for maintaining bone formation and resorption balance [37]. Direct intercellular communication between osteoblasts and osteoclasts via the extracellular matrix also plays a synergistic role in bone remodeling [35]. Therefore, we propose that excessive inhibition of RANKL expression might be detrimental to bone homeostasis, as osteoblast-driven bone formation likely requires the coordinated participation of osteoclasts. This notion offers a potential explanation for why the medium dose of OP demonstrated a stronger positive effect on the bone volume fraction in rats compared to the low dose. Overall, the medium dose of OP exhibited a better anti-osteoporotic effect, characterized by enhanced osteoblast activity, reduced osteoclast activity, and improved bone density.

Long-term DEX administration, consistent with previous findings, suppressed osteoblast activity and enhanced osteoclast activity, leading to decreased bone density and osteoporosis [38,39]. In our study, OP primarily exerted its anti-osteoporotic effects through the dual regulation of osteoblasts and osteoclasts via the Runx2 signaling pathway and the OPG/RANK/RANKL signaling pathway [40]. Further proteomic analysis of rat bone revealed that OP primarily modulates Runx2 and OPG signaling by acting on upstream pathways, specifically the PI3K-Akt signaling pathway and ECM–receptor interaction. Downregulation of the PI3K-Akt pathway in osteoblasts is linked to decreased Runx2 and OPG expression, which inhibits bone formation. Conversely, activation of the RANKL reverse signaling pathway improves bone formation by suppressing osteoclast differentiation and resorption [10,40]. Further investigation into the role of the PI3K-Akt pathway revealed that its silencing can lead to increased intracellular reactive oxygen species (ROS) and oxidative stress, contributing to mitochondrial dysfunction and osteoblast apoptosis [41,42]. The activation of the PI3K-Akt signaling pathway in our study also suggests that the EGFR may serve as an additional therapeutic target for OP [20,43,44]. The Integrin signaling pathway also warrants significant attention. Integrins, key receptors in ECM–receptor interaction, mediate cell–ECM adhesion and downstream signaling crucial for the osteogenic regulation of osteoprogenitor cells, mesenchymal stem cells, osteoblasts, and osteoclasts [43]. Indeed, PI3K-Akt acts as a critical downstream mediator of ECM–receptor interactions in osteoporosis. For example, estrogen in OVX mouse models activates Integrin αvβ3, triggering the downstream PI3K/Akt-Runx2 signaling cascade to enhance osteogenic activity and ameliorate osteoporosis [42,44]. Similarly, Icariin has been shown to promote osteogenesis by targeting Integrin α5β1, although its in vivo activity requires further investigation [45]. Osteopontin, an ECM component with an RGD sequence, is implicated in ECM–receptor interactions and exhibits a positive correlation with Runx2 and OPG levels [46,47,48]. Considering the high enrichment of ECM-related biological functions in GO and KEGG pathway analyses, OP likely modulates osteogenic and osteoclastic activity primarily through ECM–receptor interaction, activating downstream signaling pathways such as PI3K-Akt. This leads to the upregulation of Runx2 and OPG, ultimately remodeling bone structure and alleviating osteoporosis [49]. Therefore, we selected Integrins and EGFR, important common receptors in the ECM–receptor interaction and PI3K-Akt signaling pathways, for active peptide screening. Notably, EGFR can crosstalk with Integrin signaling through the PI3K, Akt, and MAPK pathways [50,51,52]. Molecular docking analysis of 144 peptides identified from oyster hydrolysates against Integrins and EGFR revealed that peptides with a length of 11 to 20 amino acid residues exhibit favorable binding characteristics, suggesting their potential anti-osteoporotic activity.

This study has certain limitations. The exclusive use of animal models means that the findings may not directly translate to human clinical outcomes. Furthermore, a deeper exploration of the underlying mechanisms is warranted. The variability between OP production batches and the complex peptide profile of OP also contribute to uncertainty in its consistent effects. Future research should prioritize the identification of key bioactive peptides and conduct more detailed mechanistic investigations to fully harness OP’s potential for osteoporosis management.

In conclusion, our study demonstrates that OP effectively mitigates DEX-induced osteoporosis by restoring the bone balance between osteoblasts and osteoclasts. The food-derived nature of these peptides, coupled with their low side effects and a scalable industrial production process, strongly supports their feasibility as a functional food for the prevention and adjuvant treatment of osteoporosis. Considering the diverse physiological activities often associated with hydrolyzed polypeptides, the strategy of exploring and expanding the functions of active peptides prepared through existing industrial processes holds significant practical relevance for the development of novel health products.

## 4. Materials and Methods

### 4.1. Materials

Alcalase 2.4L (2.4 Au) and Neutrase 0.8L (0.8 Au) were procured from Novozymes Biotechnology Co., Ltd. (Bagsværd, Denmark), and papain (1000 kU/g) was bought from Nanning Pangbo Bioengineering Co., Ltd. (Guangxi, China). Depth cartridge filters (0.6–0.8 μm and 1.5–3.0 μm) were obtained from Hangzhou Cobetter Filtration Equipment Co., Ltd. (Zhejiang, China). Dexamethasone Sodium Phosphate Injection (DEX; 5 mg/mL, H41020255) was purchased from Suicheng Pharmaceutical Co., Ltd. (Henan, China). Alendronate sodium tablets (AS; 70 mg/tablet, H20090267) were obtained from CSPC Ouyi Co., Ltd. (Hebei, China), and OG (10 g/bag, Z20003255) was obtained from Liaoning Kangchen Pharmaceutical Co., Ltd. (Liaoning, China).

### 4.2. OP Preparation

OP was prepared at the enzymatic hydrolysis workshop of Hainan Shenmeinuo Biotechnology Co., Ltd. Live oysters (*Ostrea rivularis* Gould), harvested from Lianyungang, Jiangsu Province, between November 2023 and April 2024, were shelled and rinsed to remove sediment and mucus. The cleaned oyster meat was then cut into small pieces, blanched in a boiling water bath for 5 min, and dried at 60 °C for 4 h to remove the majority of the moisture and lipids. The resulting dried meat was subsequently pulverized to a 60-mesh powder using a ball mill. Enzymatic hydrolysis was performed in a TK14 enzymatic hydrolysis tank (Yangzhou Changhai Food Machinery Co., Ltd., Jiangsu, China). The oyster powder (2000 kg) was resuspended in 20,000 L of water, and the pH was adjusted to 7.2 with solid NaOH. A mixture of proteases, consisting of 10 kg of Alcalase 2.4 L, 8 kg of Neutrase 0.8 L, and 8 kg of papain, was then added. The reaction was carried out at 60 °C for 5 h. To terminate the enzymatic reaction, the mixture was held at 90 °C for 5 h, followed by standing and filtration. A 30 mm thick defatted cotton cake was used for filtration to remove precipitates. The filtrate underwent decolorization in a TK15 decolorization tank (Yangzhou Changhai Food Machinery Co., Ltd., Jiangsu, China). It was mixed with 30 kg of activated carbon powder and stirred at 75 °C for 40 min. After decolorization, the solution was filtered through a 200-mesh plate frame, followed by microfiltration through membranes with pore sizes of 1.5–3 μm and 0.6–0.8 μm. The purified filtrate was then concentrated to 40% (*w*/*v*) using a triple-effect concentrator (Shanghai Senon Co., Ltd., Shanghai, China), followed by another microfiltration step to obtain a highly concentrated OP solution. The solution was subjected to ultra-high-temperature (UHT) treatment for 20 s and then spray-dried (Shanghai Ohkawara Dryers Co., Ltd., Shanghai, China) to yield the final OP powder, which was stored in a cool, dry place.

### 4.3. Protein Content, Amino Acid Composition, and Molecular Weight Distribution of OP

The protein content of OP was determined using a fully automated Kjeldahl nitrogen analyzer (NKY-6120, YIHON, Shanghai, China). For amino acid analysis, OP samples were first hydrolyzed according to the method of Chen et al. [53], and the amino acid content was subsequently determined using a fully automated amino acid analyzer (L-8900, Hitachi, Tokyo, Japan). The molecular weight distribution of OP was analyzed by high-performance liquid chromatography (HPLC) (Arc HPLC, Waters Co., Milford, MA, USA), following the method described by Quan et al. [12].

### 4.4. Animal Experiment Design

**Figure 7:**
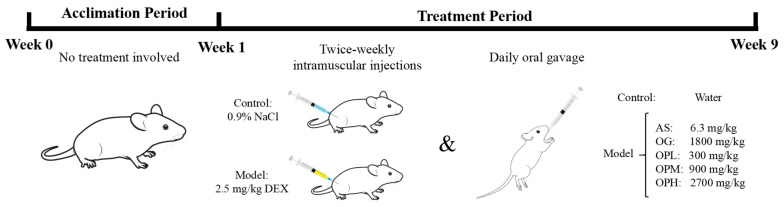
Timeline of animal experiments and treatment grouping. AS and OG served as positive controls for evaluating the anti-osteoporotic efficacy of different concentrations of OP. The image was created using free, open-source resources from SciDraw (https://scidraw.io/) and is used in accordance with their terms.

Eight-week-old male Wistar rats were purchased from Youda Biotechnology Co. (Guangzhou, China) and acclimated for one week in a dry, ventilated, SPF-grade animal facility at Guangdong Ocean University. The housing conditions were maintained at a room temperature of 22 ± 2 °C and relative humidity of 55 ± 5%. The experimental design adhered to the ARRIVE guidelines and was approved by the Laboratory Animal Ethics Committee of Guangdong Ocean University (protocol code: GDOU-LAE-2023-035). The rats were randomly divided into seven experimental groups (*n* = 12 per group): control, DEX, AS, OG, low-dose OP (OPL), medium-dose OP (OPM), and high-dose OP (OPH). Osteoporosis was induced in the latter six groups via twice-weekly intramuscular injections of 2.5 mg/kg DEX. The control group received intramuscular injections of an equivalent volume of 0.9% NaCl solution. For daily oral administration over eight weeks, the control and DEX groups received distilled water, while the remaining five groups received their respective treatments: AS (6.3 mg/kg/d), OG (1800 mg/kg/d), OPL (300 mg/kg/d), OPM (900 mg/kg/d), and OPH (2700 mg/kg/d). The Timeline of animal experiments and treatment grouping is presented in Figure 7.

Body weight was assessed weekly. After eight weeks, all rats were anesthetized with 1% sodium pentobarbital (0.4 mL/100 g, i.p.) and euthanized following a 12-h fast. Blood and various organs were collected. Organ weights (thymus, spleen, heart, lung, kidney, and liver) were measured and normalized to body weight to calculate organ indices. Blood was centrifuged at 3500 rpm for 10 min at 4 °C to separate serum, which was then stored at −80 °C for further analysis.

### 4.5. Histopathological Analysis

For liver histology, rat liver tissue samples were embedded in paraffin and sectioned after fixation. The sections were then dewaxed, rehydrated in a graded series of ethanol solutions, and stained with hematoxylin–eosin (H&E). Finally, the stained sections were observed and imaged using an optical microscope (TI-DH, Nikon, Tokyo, Japan).

Rat femurs were carefully dissected to remove muscle tissue and then decalcified in hydrochloric acid for 2 days to soften the bone matrix. The bones were subsequently fixed in 4% paraformaldehyde and embedded in paraffin using a machine (EG1150H, Leica, Wetzlar, Germany). Each femur was longitudinally sectioned into 5–6 µm thick slices using a microtome (RM2245, Leica, Wetzlar, Germany). For histological analysis, sections were dewaxed and stained with hematoxylin–eosin.

To evaluate bone mineralization, adjacent sections were stained using the Von Kossa method. Samples from each group were immersed in 1% silver nitrate solution and incubated under ultraviolet light for 20 min, followed by a 5-min rinse in 5% sodium thiosulfate solution to remove unreacted silver. 

### 4.6. Micro-Computed Tomography Analysis

The femurs of all rats were scanned using a micro-computed tomography system (micro-CT, SKYSCAN1176, Bruker, Billerica, MA, USA) at a scanning resolution of 18 µm. The X-ray source was set to 50 kV/800 µA with a 0.2 mm aluminum filter. Data were collected, and three-dimensional image reconstruction was performed using NRecon software. The trabecular bone in the region of interest was converted into a three-dimensional model. Quantitative data, including BV, TV, bone volume fraction (BV/TV), Tb.N, Tb.Th, and Tb.Sp, were then calculated using the supporting analysis software.

### 4.7. Determination of Bone Formation and Bone Absorption Indicators

Serum biochemical indicators of bone turnover were quantified using commercially available kits. Runx2 and Cath-K were analyzed with kits purchased from Cusabio (Hubei, China). OCN, RANKL, OPG, BALP, MMP-9, DPD, CTX-1, TRACP-5b, and PINP were detected using ELISA kits (Lengton Biotech, Shanghai, China). 

### 4.8. Peptidomics

OP was prepared for peptidomic analysis. Briefly, 50 mg of OP was dissolved in 500 μL of 75% methanol–water solution. The mixture was homogenized using a bead mill (35 Hz, 4 min) and sonicated in an ice bath (5 min), with the process repeated three times. After centrifugation (12,000 rpm, 4 °C, 10 min), the supernatant was collected, desalted, and dried in a vacuum concentrator at 45 °C. The resulting sample was redissolved in 50 μL of mobile phase A for LC-MS/MS analysis.

LC-MS/MS analysis was performed on an EASYnLC1200 System (Thermo Scientific, Waltham, MA, USA) equipped with a ReprosilPur 120 C18AQ column (1.9 μm, 100 μm ID × 15 cm, Dr. Maisch, Ammerbuch, Germany). The mobile phases were (A) 0.1% formic acid in 2% acetonitrile and (B) 0.1% formic acid in 80% acetonitrile. The injection volume was 4 μL, and the flow rate was 300 nL/min. Peptides were separated using the following elution gradient: 2% B from 0–2 min, ramped to 22% B at 36 min, 45% B at 56 min, 95% B at 58 min, and held at 98% B for 2 min.

Data-dependent acquisition was performed in positive mode using an Orbitrap analyzer. The MS1 scan had a resolution of 120,000 (@200 *m*/*z*) with a mass range of 350–1600 *m*/*z*. For MS2 scans, the resolution was set to 15,000 with a dynamic first mass. The top 20 most intense ions were fragmented by HCD, with a normalized collision energy of 30%. Single-charged ions and those with a charge greater than 6 were excluded from the DDA procedure. Raw mass spectrometry files were analyzed using Proteome Discoverer software (v. 2.4.0.305, Thermo Scientific, Waltham, MA, USA) against a relevant protein database.

### 4.9. Proteomics

For proteomic analysis, proteins were extracted from homogenized tissue using RIPA buffer (300 μL), followed by grinding with a bead mill (70 Hz, 4 min) and sonication in an ice-water bath (20 min). A total of 200 ng of peptides from each sample was separated and analyzed on a Vanquish Neo UHPLC system (Thermo Scientific, Waltham, MA, USA) coupled with an Astral mass spectrometer with a nano-electrospray ion source. Peptides were separated on a reversed-phase EASY-Spray™ HPLC column (150 μm × 15 cm, Thermo Scientific, Waltham, MA, USA) using a 6.9-min gradient. The mobile phases were (A) 0.1% FA in H_2_O and (B) 0.1% FA in 80% acetonitrile.

DIA was performed in profile and positive mode. MS1 scans were at a resolution of 240,000 (@200 *m*/*z*) across a mass range of 380–980 *m*/*z*. For MS2, the mass range was 150–2000, and fragmentation was carried out using HCD with a normalized collision energy (NCE) of 25% and an isolation window of 2 *m*/*z*. Raw files were imported into Spectronaut software (v. 18.2.230802.50606; Biognosys AG, Zurich, Switzerland) for database searching and subsequent qualitative analysis.

### 4.10. Molecular Docking Simulation

Discovery Studio software (version 2017 R2, Biovia, San Diego, CA, USA) was used to evaluate the potential osteogenic activity-promoting mechanism of OP. Firstly, in the Small Molecules module, energy minimization (Full Minimization) and Prepare Ligands preprocessing were performed on peptide ligands. Subsequently, the receptor proteins were optimized: Integrins α5β1 (PDB ID: 3VI4) and αvβ3 (PDB ID: 1L5G) were processed to remove water molecules and add hydrogen via the Macromolecules module, and the Prepare Protein structure optimization was completed. Based on the classic RGD (Arg-Gly-Asp) interaction sites (Site 2 of 3VI4 and Site 1 of 1L5G), the binding sites were defined using the Define and Edit Binding Site module (the original ligands were removed, and the radius was set to 15r). After undergoing the same preprocessing, the binding site of EGFR (PDB ID: 1IVO) was defined according to the active center residues reported in the literature. Finally, the CDOCKER algorithm in the Receptor–Ligand Interactions module was used for semi-flexible docking between the above three targets and peptide ligands. The results were visualized by Discovery Studio, and the binding modes were analyzed.

### 4.11. Statistical Analysis

Statistical analysis was conducted using SPSS 22.0 software (SPSS Inc., Armonk, NY, USA). Results are expressed as the mean ± standard deviation. One-way analysis of variance (ANOVA) and Student’s *t*-test were used for statistical comparison. A probability value of *p* < 0.05 was considered statistically significant.

## Figures and Tables

**Table 1:** Potential anti-osteoporotic active peptides with lengths of 11–20 amino acid residues.

Peptide Sequence	Length	Molecular Mass (Da)	Bioactive Score	Toxicity	Binding Energy (kcal/mol)		
					Integrins α5β1	Integrins αvβ3	EGFR
GQKDSYVGDEAQSKRGILT	19	2052.03	0.2472	Nontoxic	295.89	240.23	272.95
GQKDSYVGDEAQSKRGILTL	20	2165.12	0.2789	Nontoxic	290.02	138.42	281.91
KDAENRATEAERTVSKL	17	1918.00	0.0956	Nontoxic	274.91	246.31	252.32
TTTAEREIVRDIKEK	15	1788.98	0.0291	Nontoxic	270.52	246.22	253.89
TTAEREIVRDIKEK	14	1687.93	0.0333	Nontoxic	242.33	225.85	236.60
SYVGDEAQSKRGIL	14	1522.79	0.3127	Nontoxic	226.72	208.52	203.97
DLAGRDLTDYLMKIL	15	1736.93	0.3487	Nontoxic	225.92	233.64	225.00
TAEREIVRDIKEK	13	1586.89	0.0446	Nontoxic	225.29	225.06	226.24
AEREIVRDIKE	11	1357.74	0.0588	Nontoxic	223.52	190.44	206.26
DVDIRKDLYAN	11	1321.67	0.1576	Nontoxic	219.93	179.26	207.68
AEREIVRDIKEK	12	1485.84	0.0673	Nontoxic	212.54	225.03	215.12
DLAGRDLTDYLMK	13	1510.76	0.3423	Nontoxic	209.64	208.75	198.61
KQEYDESGPSIVH	13	1488.70	0.1903	Nontoxic	209.09	197.81	190.45
DLAGRDLTDYL	11	1251.62	0.2583	Nontoxic	204.29	191.17	189.01
DLAGRDLTDYLM	12	1398.66	0.3555	Nontoxic	203.28	212.97	180.42
KSYELPDGQVITIG	14	1519.80	0.2720	Nontoxic	199.99	140.55	194.27
KQEYDESGPSIV	12	1351.64	0.1908	Nontoxic	196.70	186.03	171.52
EYDESGPSIVHR	12	1388.64	0.1670	Nontoxic	188.00	152.92	166.65
LESSTAGGVAS	11	978.47	0.0860	Nontoxic	169.15	154.10	142.23

## Data Availability

Data are contained within the article and Supplementary Materials.

## Supplementary Materials

The following supporting information can be downloaded at https://www.mdpi.com/article/10.3390/md23090356/s1: Table S1: Effects of AS, OG, and OP on body weight in DEX-induced osteoporotic rats; Table S2: Differentially expressed genes in the OPH group versus the DEX group; Table S3: Polypeptides show binding affinity for receptors Integrins α5β1 (1L5G), Integrins αvβ3 (3VI4), or EGFR (1IVO) in OP.

## Abbreviations

The following abbreviations are used in this manuscript:

AS	Alendronate Sodium
BALP	Bone Alkaline Phosphatase
BV	Bone Volume
Cath-K	Cathepsin K
CTX-1	C-Terminal Telopeptide of Type I Collagen
DEX	Dexamethasone, Dexamethasone Sodium Phosphate
DIA	Data-Independent Acquisition
DPD	Deoxypyridine
EGFR	Epidermal Growth Factor Receptor
MMP-9	Matrix Metallopeptidase 9
OCN	Osteocalcin
OG	*Gushukang Granules*, Osteoporosis Granules
OP	Oyster Peptide
OPG	Osteoprotegerin
OPH	High-Dose Oyster Peptide
OPL	Low-Dose Oyster Peptide
OPM	Medium-Dose Oyster Peptide
PINP	N-Terminal Propeptides of Procollagen Type Ⅰ
ROS	Reactive Oxygen Species
Runx2	Runt-Related Transcription Factor 2
Tb.N	Trabecular Number
Tb.Sp	Trabecular Separation
Tb.Th	Trabecular Thickness
TCM	Traditional Chinese Medicine
TRACP-5b	Tartrate-Resistant Acid Phosphatase 5b
TV	Tissue Volume
OVX	Ovariectomy
