# Supplementary material for: Improvement Effect and Regulation Mechanism of Oyster Peptide on Dexamethasone-Induced Osteoporotic Rats
Source: Mar Drugs. 2025 Sep 11;23(9):356. doi: 10.3390/md23090356 (PMC12472170; doi:10.3390/md23090356)
Supplement: Supplementary file 1 [file marinedrugs-23-00356-s001.zip › marinedrugs-3842758-supplementary.pdf]

Table S1. Effect of AS, OG and OP on body weight in DEX-induced osteoporotic rats

Week	Control	DEX	AS	OG	OPL	OPM	OPH
0	355.71 ± 22.08	344.44 ± 15.72	343.76 ± 19.73	342.03 ± 19.17	354.4 ± 16.58	352.33 ± 17.44	355.64 ± 21.58
1	366.11 ± 24.15	355.96 ± 14.72	357.99 ± 19.71	347.43 ± 16.47	365.55 ± 19.33	363.44 ± 19.68	369.08 ± 16.21
2	376.53 ± 27.32	345.48 ± 13.64	347.48 ± 19.15	343.58 ± 15.8	355.59 ± 20.8	352.97 ± 16.49	356.84 ± 19.02
3	381.51 ± 26.72	340.25 ± 13.5	346.29 ± 20.37	340.79 ± 16.98	354.25 ± 20.28	350.36 ± 18.1	353.56 ± 18.98
4	393.31 ± 25.55	337.09 ± 16.02	345.05 ± 20.39	340.18 ± 17.14	344.92 ± 42.36	352.14 ± 19.52	355.96 ± 18.49
5	403.16 ± 28.63	343.21 ± 15.04	353.83 ± 20.54	344.88 ± 18.29	359.89 ± 21.2	360.07 ± 21.75	362.23 ± 16.6
6	413.74 ± 30.28	345.55 ± 13.51	356.74 ± 20.39	351.43 ± 19.39	355.51 ± 31.18	363.42 ± 22.71	365.82 ± 16.51
7	419.8 ± 30.51	350.58 ± 14.79	358.65 ± 22.07	354.26 ± 20.95	363.39 ± 23.36	367.9 ± 23.81	369.18 ± 15.91
8	425.06 ± 32.19	344.79 ± 15.47	362.66 ± 21.22	358.9 ± 22.82	367.06 ± 24.65	365.59 ± 23.51	369.78 ± 14.68
9	430.23 ± 33.53	350.46 ± 14.57	363.66 ± 23.54	360.04 ± 25.06	367.66 ± 25.09	373.64 ± 23.6	374.4 ± 16.02

Table S2. Differentially expressed genes in the OPH group versus the DEX group

GeneName	P-value	log_2(Fold Change)	GeneName	P-value	log_2(Fold Change)
Spink1l	0.10243	-9.475	Tns1	0.04359	0.302
Tubgcp3	0.00226	-8.617	rCG_45963	0.00203	0.314
Bag2	0.13361	-8.306	S100a10	0.04950	0.321
Dhrsx	0.05638	-6.887	Fermt2	0.00902	0.339
Larp4	0.17068	-6.716	S100a4	0.01271	0.349
Tbc1d5	0.00099	-3.654	Eif3j	0.01573	0.359
Tomm5	0.00570	-2.796	Lama4	0.01158	0.366
Cyp4f18	0.02289	-2.273	Ccdc102a	0.02546	0.366
B4galt1	0.00334	-1.498	Cavin3	0.04353	0.375
Parp4	0.01473	-1.145	Clu	0.00467	0.395
Tram1	0.04706	-1.069	S100a6	0.00261	0.397
Fbln5	0.03898	-1.043	Sh3bp1	0.04969	0.423
Tra2b	0.01446	-0.852	Osbpl11	0.00773	0.424
Tk1	0.00499	-0.782	Hrg	0.00680	0.428
Hddc2	0.00239	-0.763	Lum	0.00843	0.431
Nudt16l1	0.04128	-0.666	Xrcc5	0.01681	0.438
Lrrc47	0.02713	-0.640	C4	0.00182	0.439
U2af2	0.02655	-0.601	Col15a1	0.03534	0.459
Abcc4	0.03157	-0.503	Bgn	0.04871	0.459
Arfgef1	0.03683	-0.493	C4b	0.02121	0.463
rCG_29759	0.03025	-0.445	Vim	0.01822	0.467
Tnn	0.02567	-0.442	Klc1	0.00353	0.493
Dnmt1	0.02489	-0.440	rCG_42279	0.01936	0.495
Txn1	0.04543	-0.439	Kng2	0.04323	0.496
Capns1	0.01612	-0.424	Pgp	0.03575	0.503
rCG_41494	0.00395	-0.421	ENSRNOG00000066904	0.01125	0.524
Dnajb6	0.02972	-0.416	Smarca4	0.00602	0.525
Lars1	0.03601	-0.393	Ago2	0.00337	0.532
Gfm1	0.00128	-0.384	Kng1	0.00795	0.536
Rpl15l3	0.02205	-0.383	Atp5a1	0.01193	0.539
Rpl32	0.01023	-0.376	Olfml1	0.03498	0.548
Echdc1	0.03381	-0.374	Snrpf	0.00714	0.563
Golt1b	0.03813	-0.353	Fbln1	0.00369	0.575
Hook3	0.00143	-0.338	Eif2s2	0.02456	0.581
Smok2a	0.03732	-0.303	Comp	0.03618	0.594
Atad3a	0.01201	-0.282	Hrg	0.03651	0.598
Plac8	0.01645	-0.281	Prelp	0.00176	0.615
Lrpap1	0.02970	0.277	Ppil1	0.02503	0.616
Itih4	0.04147	0.278	Meltf	0.04406	0.624
A1i3	0.03290	0.283	Pter	0.03340	0.624
Ubac1	0.03586	0.290	Zmym4	0.00566	0.628
Cfi	0.03981	0.292	Ackr1	0.04228	0.631

Adjusted p-value < 0.05.

The text continues here (Table S2)

GeneName	P-value	log2(Fold Change)
Tgfbi	0.00639	0.649
Dcn	0.02332	0.650
Ecm1	0.03494	0.654
Col14a1	0.00070	0.664
Fmod	0.01147	0.689
Tubb2a	0.03198	0.692
Tnc	0.02263	0.697
Gmppa	0.04626	0.715
Agt	0.00145	0.734
Safb	0.04690	0.792
Sod3	0.00017	0.810
Crip1	0.00914	0.812
Igkvl16	0.04010	0.815
Tnxb	0.02061	0.823
rCG_47614	0.04881	0.823
Mfge8	0.00037	0.850
Thbs3	0.01825	0.893
Rbks	0.01471	0.905
Atp5a1	0.01111	0.929
Ehbp1l1	0.01194	0.936
Atp5if1	0.01308	0.943
ENSRNOG00000063148	0.02098	0.952
Col6a6	0.01375	0.998
Eif2ak2	0.02946	1.008
Fndc1	0.02447	1.035
Cmas	0.01460	1.153
Tnxb	0.01245	1.201
Ddi2	0.04635	1.433
Rab12	0.04919	1.523
Hmgn2	0.01814	1.591
Fip1l1	0.00412	2.020
Eif4ebp1	0.04676	2.060
Ptpn23	0.03083	2.120
Serpina3c	0.02403	2.149
Smndc1	0.02501	2.201
Rai1	0.02784	2.472
Kras	0.00724	2.924
Clasp1	0.00302	3.493
Chi3l3	0.07677	9.956

Table S3. Polypeptides show binding affinity for receptors Integrins $\alpha5\beta1$ (1L5G), Integrins $\alpha v\beta3$ (3VI4) or EGFR (1IVO) in OP.

Length	Peptide Sequence	Bioactive Score	Binding energy (kcal/mol)		
			Integrins $\alpha5\beta1$: 1L5G	Integrins $\alpha v\beta3$: 3VI4	EGFR: 1IVO
6	EAKVLE	0.0486	144.95	139.84	127.41
6	EGLDVH	0.0919	141.79	125.12	113.75
6	QTFAEL	0.2309	124.25	129.27	106.60
6	KSEPNI	0.1515	127.12	124.26	99.27
6	GLDVHL	0.3075	115.72	111.18	105.86
6	AGFAGD	0.4559	110.92	111.40	94.81
6	LTEAPL	0.1649	108.50	104.08	86.01
6	VPIYEG	0.2059	112.52	101.59	84.29
6	GVMVGM	0.4326	96.43	100.58	89.85
6	GLDNPL	0.6345	108.72	94.00	81.96
6	PVLLTE	0.1005	110.49	96.47	77.31
6	IGGSIL	0.3906	91.90	108.57	78.35
6	PSTVDI	0.1344	104.05	101.57	72.53
6	FPGIAD	0.5613	95.81	89.05	83.22
6	GQPLLV	0.4844	90.31	92.32	77.40
6	LDNPLP	0.6267	97.84	85.27	73.05
6	LPVGNL	0.3871	83.69	87.91	77.57
6	FPSIVG	0.6291	85.18	89.84	71.92
6	VPVLLG	0.2984	90.22	83.86	69.75
6	PVLLGG	0.3998	87.21	82.38	68.03
7	AEREIVR	0.0989	152.96	139.09	134.01
7	TERGYSF	0.2088	139.26	120.90	115.07
7	ELPDGQV	0.1192	135.51	123.99	112.61

All peptides are nontoxic.

The text continues here (Table S3)

Length	Peptide Sequence	Bioactive Score	Binding energy (kcal/mol)		
			Integrins $\alpha 5\beta 1$: 1L5G	Integrins $\alpha v\beta 3$: 3VI4	EGFR: 1IVO
7	GQVITIG	0.1725	114.06	122.74	98.90
7	IGGSILA	0.2908	105.48	108.92	90.22
7	APEEHPV	0.1836	113.34	95.47	94.84
7	LQSSPLP	0.5004	84.38	79.02	74.58
7	VDNGSGM	0.2531	/	117.89	111.21
8	LEEDMERS	0.0728	184.92	157.12	155.53
8	LDLAGRDL	0.3071	159.10	126.34	136.10
8	LESSTAGG	0.1368	141.46	133.02	118.72
8	TEAPLNPK	0.2210	132.13	123.62	112.05
9	DDMEKIWHH	0.3679	193.98	156.18	163.37
9	AEREIVRDI	0.0813	186.49	156.69	149.58
9	GSIVEESHA	0.0745	165.35	157.52	139.91
9	GFAGDDAPR	0.5989	155.26	145.97	128.73
10	LDLAGRDLTD	0.1373	182.42	144.84	152.52
10	AGFAGDDAPR	0.5619	167.53	155.06	137.33
10	AITEVDLERA	0.0789	195.72	167.20	164.82
10	ANREKMTQIM	0.1623	192.03	/	150.45
10	GFAGDDAPRA	0.5884	165.49	/	142.29
11	AEREIVRDIKE	0.0588	223.52	190.44	206.26
11	DVDIRKDLYAN	0.1576	219.93	179.26	207.68
11	DLAGRDLTDYL	0.2583	204.29	191.17	189.01
11	LESSTAGGVAS	0.0860	169.15	154.10	142.23
11	LDLAGRDLTDY	0.1585	201.83	/	175.57

All peptides are nontoxic.

The text continues here (Table S3)

Length	Peptide Sequence	Bioactive Score	Binding energy (kcal/mol)		
			Integrins α5β1：1L5G	Integrins αvβ3：3VI4	EGFR：1IVO
11	TLRNLFGNNKL	0.2669	165.92	/	155.10
12	AEREIVRDIKEK	0.0673	212.54	225.03	215.12
12	DLAGRDLTDYLM	0.3555	203.28	212.97	180.42
12	KQEYDESGPSIV	0.1908	196.70	186.03	171.52
12	EYDESGPSIVHR	0.1670	188.00	152.92	166.65
12	GIVLDSGDGVSH	0.1900	209.29	/	175.81
13	TAEREIVRDIKEK	0.0446	225.29	225.06	226.24
13	DLAGRDLTDYLMK	0.3423	209.64	208.75	198.61
13	KQEYDESGPSIVH	0.1903	209.09	197.81	190.45
14	TTAEREIVRDIKEK	0.0333	242.33	225.85	236.60
14	SYVGDEAQSKRGIL	0.3127	226.72	208.52	203.97
14	KSYELPDGQVITIG	0.2720	199.99	140.55	194.27
14	GFAGDDAPRAVFPS	0.5710	171.19	/	/
15	TTTAEREIVRDIKEK	0.0291	270.52	246.22	253.89
15	DLAGRDLTDYLMKIL	0.3487	225.92	233.64	225.00
15	SYVGDEAQSKRGILT	0.2330	230.17	/	216.57
17	KDAENRATEAERTVSKL	0.0956	274.91	246.31	252.32
18	GQKDSYVGDEAQSKRGIL	0.3272	/	276.06	257.27
18	TEAPLNPKANREKMTQIM	0.3922	211.37	/	220.56
19	GQKDSYVGDEAQSKRGILT	0.2472	295.89	240.23	272.95
20	GQKDSYVGDEAQSKRGILTL	0.2789	290.02	138.42	281.91
21	VSSAGTLSSYGQVVGSNVYSA	0.4684	223.19	231.77	225.53
22	STQEGRLRDGTHDKLYSYGGRG	0.3850	/	/	307.27

All peptides are nontoxic.

The text continues here (Table S3)

Length	Peptide Sequence	Bioactive Score	Binding energy (kcal/mol)		
			Integrins $\alpha5\beta1$: 1L5G	Integrins $\alpha v\beta3$: 3VI4	EGFR: 1IVO
23	DVRGVMVEYLEVYSINANSNEVV	0.0215	265.69	/	275.86
24	TGIVLDSGDGVSHTVPIYEGYALP	0.1191	153.82	/	245.85
24	TKEITETAFPFRMVALDMNGEPVQ	0.0399	/	/	267.38
26	TKEITETAFPFRMVALDMNGEPVQAL	0.0187	/	/	284.82
26	ESFARIHNGGPRGCRNSNTEGYWRRV	0.6549	/	/	259.36
28	HSFLSDTPVTSLTMSVPVLRHPHVYHAF	0.1112	132.61	122.65	221.95
28	QQWPIAVCAEHKSCFIPDSVVGWGIHGL	0.4307	259.29	/	264.69
28	RTTGIVLDSGDGVSHTVPIYEGYALPHA	0.1299	/	/	283.73
28	VLSGGTTMFPGIADRMQKEVTALAPPTM	0.0507	/	/	/
29	DLAGRDLTDYLMKILTERGYSFTTTAERE	0.2589	/	/	217.35
29	AEHKSCFIPDSVVGWGIHGLWPSSDTESK	0.2448	/	212.03	/
29	PFPSISSPDFARCSLKSPSSTNMVKSEPN	0.1799	/	/	211.72
30	EPVGIPDVRGVMVEYLEVYSINANSNEVVS	0.0419	/	266.99	/
30	TKEITETAFPFRMVALDMNGEPVQALAANE	0.0124	/	/	238.63
32	QGILPSKTAGYMINETEAAVKRELGVDAVIEC	0.1163	/	/	221.15
33	GTHDKLYSYGGRGFSVLRSDTMERIYDSGSIVE	0.0862	/	/	337.83
42	QIMFETFNSPAMYVAIQAVLSLYASGRTTGIVLDSGDGVSHT	0.0265	/	/	437.60

All peptides are nontoxic.
